# Intra-articular collagenase in the spinal facet joint induces pain, DRG neuron dysregulation and increased MMP-1 absent evidence of joint destruction

**DOI:** 10.1038/s41598-020-78811-3

**Published:** 2020-12-15

**Authors:** Meagan E. Ita, Prabesh Ghimire, Rachel L. Welch, Harrison R. Troche, Beth A. Winkelstein

**Affiliations:** 1 Department of Bioengineering, University of Pennsylvania, 210 S. 33rd Street, 240 Skirkanich Hall, Philadelphia, PA 19104-6392 USA; 2 Department of Neurosurgery, University of Pennsylvania, Philadelphia, PA 19104 USA

**Keywords:** Neuropathic pain, Musculoskeletal system

## Abstract

Degeneration is a hallmark of painful joint disease and is mediated by many proteases that degrade joint tissues, including collagenases. We hypothesized that purified bacterial collagenase would initiate nociceptive cascades in the joint by degrading the capsular ligament’s matrix and activating innervating pain fibers. Intra-articular collagenase in the rat facet joint was investigated for its effects on behavioral sensitivity, joint degeneration, and nociceptive pathways in the peripheral and central nervous systems. In parallel, a co-culture collagen gel model of the ligament was used to evaluate effects of collagenase on microscale changes to the collagen fibers and embedded neurons. Collagenase induced sensitivity within one day, lasting for 3 weeks (p < 0.001) but did not alter ligament structure, cartilage health, or chondrocyte homeostasis. Yet, nociceptive mediators were increased in the periphery (substance P, pERK, and MMP-1; p ≤ 0.039) and spinal cord (substance P and MMP-1; p ≤ 0.041). The collagen loss (p = 0.008) induced by exposing co-cultures to collagenase was accompanied by altered neuronal activity (p = 0.002) and elevated neuronal MMP-1 (p < 0.001), suggesting microscale collagen degradation mediates sensitivity in vivo. The induction of sustained sensitivity and nociception without joint damage may explain the clinical disconnect in which symptomatic joint pain patients present without radiographic evidence of joint destruction.

## Introduction

Chronic pain poses a substantial public health challenge, with 18% of Americans reporting pain interfering with daily life and annual costs in the United States exceeding those for cancer and diabetes combined^1,2^. A primary source of chronic pain is joint disease associated with osteoarthritis^1,3^. Neck and low back pain are among the most prevalent chronic syndromes^1^, and can result from pathology of the spinal facet joints which are susceptible to trauma^4^ and degeneration^5^. Innervated joint tissues, like the capsular ligament that surrounds the spinal facet^6^, facilitate pain transmission. Since nerve fibers and resident fibroblast-like synoviocytes (FLS) in the facet capsule are mechanosensitive^7,8^, load-induced disruption of the capsule’s Type I collagen network can initiate pathological cellular cascades^9^. Collagen fiber deformation also activates nociceptive afferents^10,11^, which have their cell bodies in the dorsal root ganglia (DRG) and synapse with spinal dorsal horn neurons to transmit noxious stimuli^12^.

Degeneration is a hallmark of painful joint disease and is mediated by a host of proteolytic enzymes that degrade joint tissues. Collagenases, for example, are matrix metalloproteinases (MMPs) that degrade the extracellular matrix (ECM) of the cartilage and/or capsular ligament, altering joint structure and mechanics^13,14^. The interstitial collagenases, MMP-1, -8, and -13, and the transmembrane protein MMP-14, degrade triple helical collagen^13^. MMP-1 protein levels increase in the joint capsule after elbow trauma^15^ and with facet degeneration^16^, and MMP-1 concentration increases in synovial fluid after knee trauma^17^. Intra-articular injection of MMP-1 and MMP-13 in hamster knees produces collagen fragments indicative of Type I collagen cleavage within even 15 minutes^18^, demonstrating that interstitial collagenases can quickly initiate collagen damage. Collagenase-injected knees also exhibit increased laxity^19^, suggesting decreased biomechanical integrity. Since the facet capsular ligament is primarily triple helical Type I collagen, it is especially susceptible to degradation by collagenases^13^. Although it is possible that pathological levels of collagenases could trigger nociceptive cascades in the afferents embedded in the capsule’s collagen network via degradation of the fibers surrounding the nerves , the mechanistic involvement of collagenases in degenerative joint pain is unclear.

There is a known discordance between radiographic signs of facet joint degeneration and pain symptoms^20^, with some studies reporting positive relationships between pain and evidence of joint degeneration^5,21^, and others finding weak or no correlations between damage and symptoms^22,23^. Animal studies injecting intra-articular bacterial collagenase find more severe joint degeneration with higher doses and longer time post-injection^24–26^. Further, the progression and extent of joint damage depends on the *type* of bacterial collagenase. Most studies use a crude bacterial collagenase that also contains high levels of secondary proteases like trypsin and clostripain; intra-articular crude bacterial collagenase in the knee and lumbar facet joints in animal models mimics the clinical scenario in which joint damage, particularly to cartilage and bone, is severe^5,21,24–26^. Crude collagenases induce chondrocyte disorganization, cartilage thinning and fibrillation, subchondral bone defects, and proteoglycan loss^24–26^. Movement-induced pain-like behaviors and tactile allodynia are evident as early as 3 days after injection lasting for 8 weeks^25,26^, and are hypothesized to be mediated by the severe progression of joint damage and subsequent exposure of nerve fibers in eroded subchondral bone^25^.

Although studies of intra-articular crude collagenase posit a mechanism by which structural damage may mediate sensitivity^25,26^, that mechanism does not explain clinical cases in which patients do not present with imaging evidence of joint destruction^22,23^. As such, it is possible that in some cases of chronic joint pain, nociception may be mediated by mechanisms *other* than structural damage detected radiographically. Given the elevated levels of interstitial collagenases in joint disease^15–17^, and the potential for the innervated capsular ligament to undergo collagen degradation^13^, we hypothesize that bacterial collagenase may initiate nociceptive cascades in joint afferents by changing the capsule’s collagen network, which alters the local microstructural environment of afferents. Indeed, injecting *purified* bacterial collagenase, which lacks the proteolytic enzymes capable of degrading cartilage, induces increased joint laxity and suggests that collagenase-mediated degradation of Type I collagen-rich tissues, like the capsular ligament, can destabilize the joint and trigger pathological mechanotransduction cascades^27^. Although that study supports the notion that collagenase-mediated collagen degradation alone might play a role in joint disease, effects on pain were not investigated.

We tested whether intra-articular purified bacterial collagenase in cervical facets induces pain-like behaviors and investigated degenerative and nociceptive pathways in the peripheral and central nervous systems in the rat. We quantified behavioral sensitivity by measuring mechanical hyperalgesia, which is a component of the complex human experience of pain in the human^28^. Mechanical hyperalgesia was measured for 21 days after bilateral injection into the C6/C7 cervical facets. A 60U dose of bacterial collagenase was chosen based on prior reports using crude bacterial collagenase injected into lumbar facet joints that found severe joint degradation accompanied by tactile allodynia^24,25^, and that same dose increasing neuronal expression of biochemical regulators of injury and nociception in DRG neurons^29^. Effects on joint structure were quantified using histology of the capsular ligament and cartilage at day 21, a timepoint that allows for any detectable joint damage to develop^24^. Hypoxia-inducible factor 1α (HIF-1α), a protein that regulates chondrocyte survival in pathologically hypoxic environments, was also probed for its role in homeostatic maintenance in the cartilage^30,31^. Substance P, a neuropeptide involved in nociception^7,12^, and phosphorylated ERK (pERK), a mitogen-activated kinase indicative of noxious injury^32,33^, were investigated in the DRG and spinal cord. We also assayed MMP-1 protein responses in the DRG and spinal cord given its emerging role in joint pain and in diseased joints with severe-to-very mild degeneration^15–17^. Since collagenase digestion alters fiber kinematics^29^ and we hypothesize that intra-articular collagenase changes the capsular ligament’s ECM, we mimicked intra-articular collagenase exposures using an in vitro co-culture collagen gel model of the capsular ligament^10^ and quantified the effect of collagenase exposure on the amount of collagen, its network microstructure, live-neuronal signaling responses, and the same neuronal MMP-1 as characterized in vivo. The co-culture model minimizes the confounding factors present with in vivo injections and enables measuring fiber- and cell-level outcomes.

## Results

### Intra-articular bacterial collagenase induces immediate and sustained behavioral sensitivity absent overt joint damage

**Figure 1:**
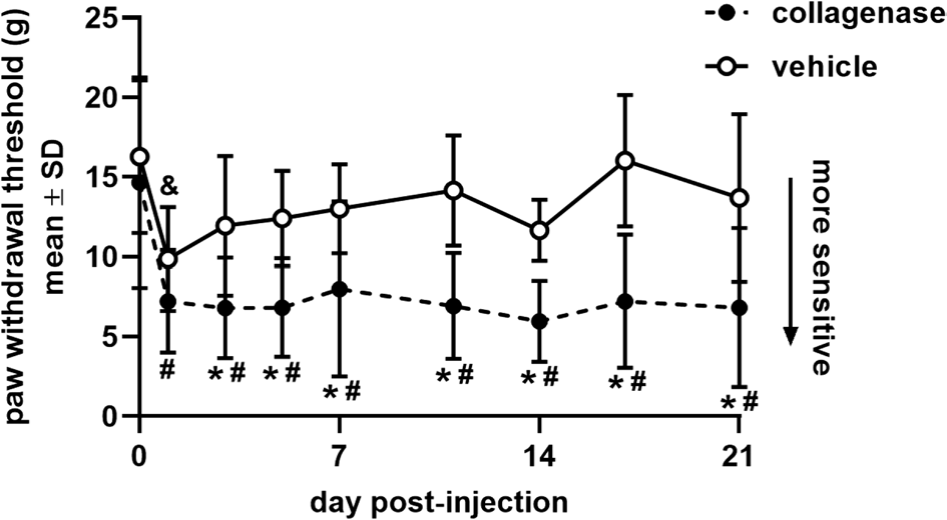
Paw withdrawal threshold (PWT) for 21 days after intra-articular injection of collagenase or vehicle. PWTs quantifying mechanically evoked pain-like behavior exhibit a decrease (corresponding to greater sensitivity) after collagenase injection (12 rats) at all days (#p < 0.001) compared to baseline (day 0). Intra-articular injection of vehicle (6 rats) produces a decrease in PWT from baseline (&p = 0.009) at day 1 that resolves by day 3. Starting on day 3, intra-articular collagenase induces sensitivity that is significantly lower (*p ≤ 0.036) than vehicle for all days of testing. All p-values are calculated using a repeated-measures ANOVA and post-hoc Tukey HSD tests; data are expressed as mean ± standard deviation (SD) for each group.

To assess whether intra-articular bacterial collagenase induces pain-like behaviors, behavioral sensitivity was quantified before, and for 21 days after, a single injection of purified bacterial collagenase (60U) into the bilateral C6/C7 facet joints of male Holtzman rats^34^. Since the rat C6/C7 dermatome extends to the forepaw^35^, paw withdrawal threshold (PWT) was measured to quantify mechanical hyperalgesia^34^. Collagenase decreases the PWT from baseline un-injected responses as early as one day after its injection (p < 0.001), with collagenase-injected rats exhibiting increased sensitivity and significantly (p < 0.001) decreased PWT for the full 21-day period after injection (Fig. 1). Although a bilateral injection of saline vehicle also produces an initial decrease in PWT on day 1 (p = 0.009), that decrease does not persist (Fig. 1); the PWT of collagenase-injected rats is significantly lower (p ≤ 0.036) than the vehicle group starting on day 3 and lasting for all days after (Fig. 1).

**Figure 2:**
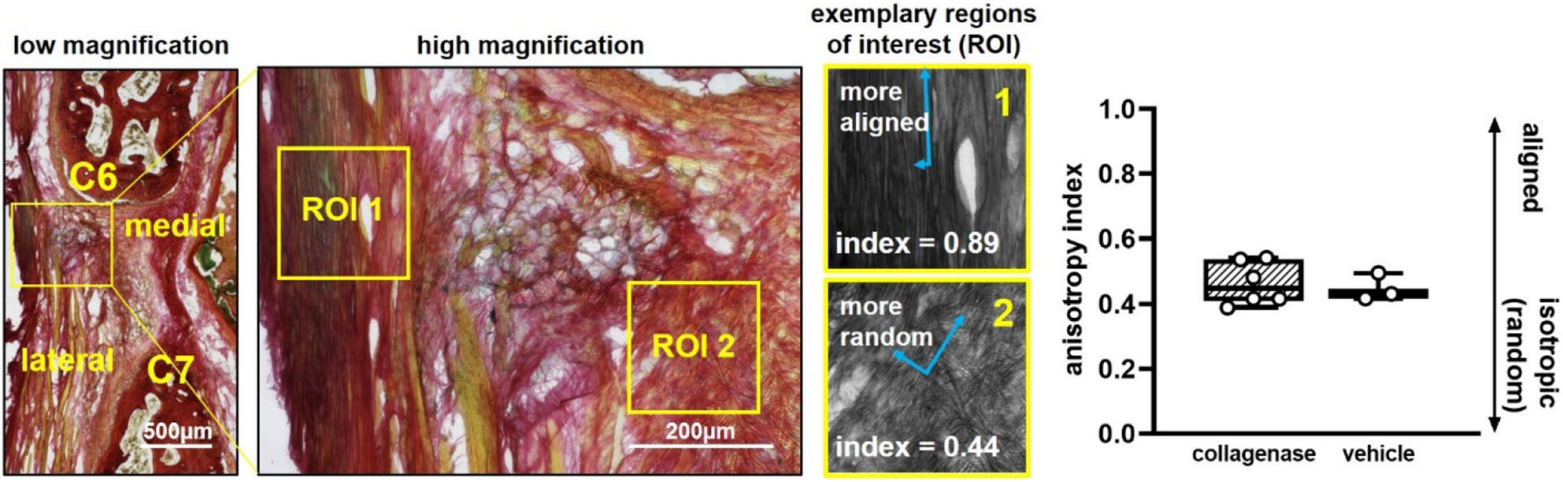
Collagen fiber orientation in the capsular ligament at 21 days after intra-articular injection of collagenase or vehicle. A low magnification image of collagen fibers stained with Picrosirius Red/Alcian Blue depicts the anatomical orientation of a C6/C7 joint. A high magnification image of the capsule in the lateral-to-middle region shows two separate regions of interest (ROIs) from a sample after collagenase injection with principal orientation axes (blue arrows) and their anisotropy indices (white text) overlaid. ROI 1 demonstrates collagen fibers with highly aligned orientation and ROI 2 demonstrates fibers with a less aligned, more isotropic orientation. Intra-articular collagenase (6 joints) does not change the orientation of collagen networks from vehicle (3 joints) (two-tailed t-test; p = 0.731). Box-and-whisker plots show horizontal lines representing the first (lower) quartile, median, and third (upper) quartile of the data; whiskers represent the minimum and maximum of the data set. Data points for individual joints are superimposed on boxplots and are the mean value quantified using 5–6 images/rat and 2–4 ROIs/image.

**Figure 3:**
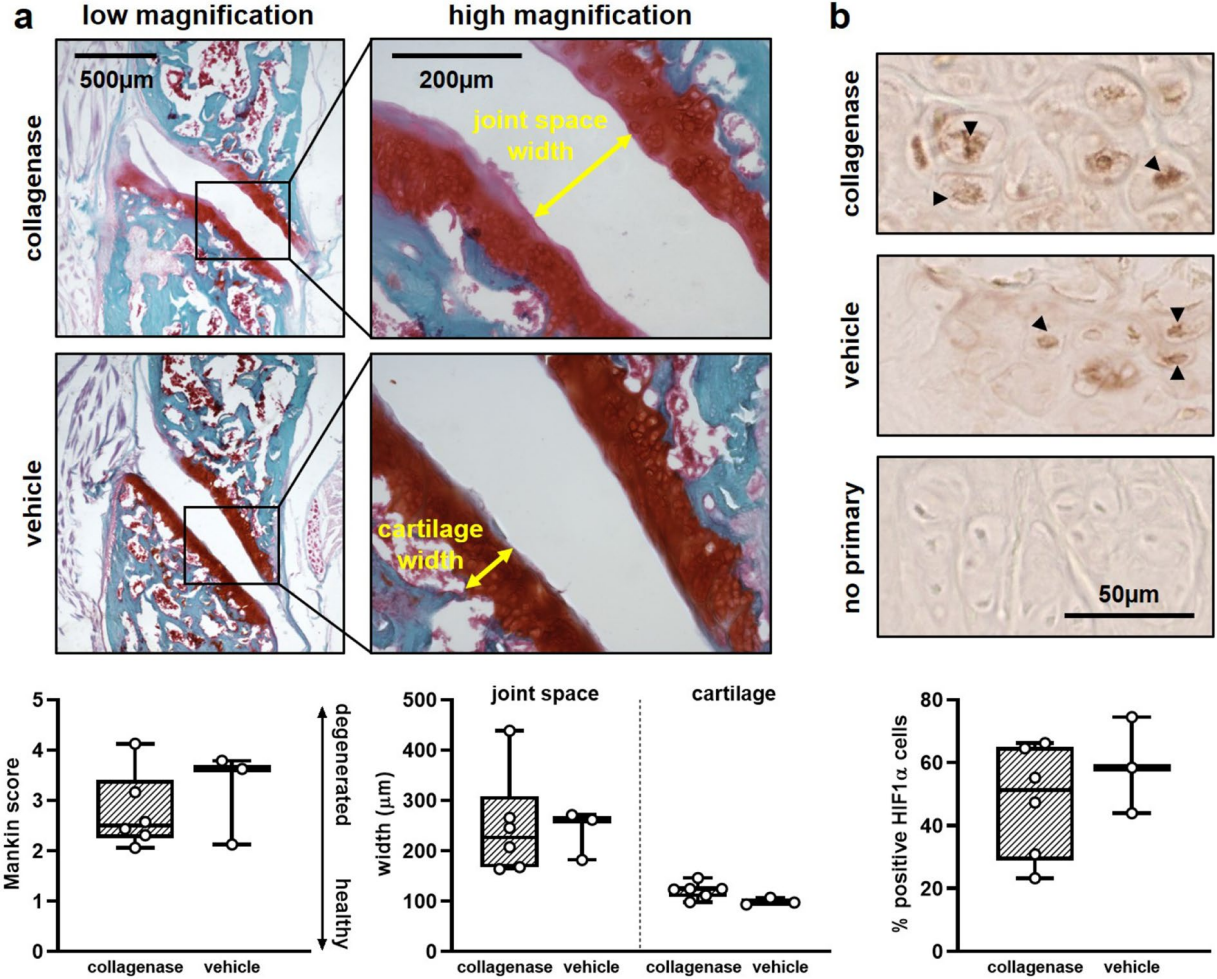
Assessment of damage and health in the facet joint’s cartilage, bone, and articular chondrocytes at 21 days post-injection. (**a**) Low and high magnification images of bone and cartilage staining with Safranin O/Fast Green reveals no significant difference in Mankin score (Wilcoxon test; p = 0.444); a mild decrease in cartilage staining and slight fibrillation is observed in some collagenase-injected joints. Similar to the Mankin score, neither joint space width (two-tailed t-test; p = 0.841) nor cartilage width (two-tailed t-test; p = 0.111) change after intra-articular collagenase (6 joints) compared to intra-articular vehicle (3 joints). (**b**) Images show chondrocytes in the articular cartilage immunolabeled with HIF-1α where black arrowheads indicate positive HIF-1α labeling. For each image, HIF-1α is quantified in regions that are four times larger than those shown in **b**; zoomed images are shown here to better visualize chondrocyte labeling. Chondrocytes exhibit a similar pattern of positive labeling regardless of group (collagenase n = 6; vehicle n = 3) that is not different (two-tailed t-test; p = 0.423). An image of the no primary, negative control for the HIF-1α label is also shown. Box-and-whisker plots show horizontal lines representing the first (lower) quartile, median, and third (upper) quartile of the data. Whiskers represent the minimum and maximum of the data set. Data points for individual joints are superimposed on boxplots and show the mean value from 6–8 images/rat for the Mankin score, 3–4 images/rat for the joint space and cartilage width measurements, and 5 images/rat for the HIF-1α labeling.

Since intra-articular collagenase induces sustained pain-like behavioral sensitivity (Fig. 1) and sensitivity is reported to parallel joint damage^25^, we assayed the structural integrity and cellular homeostasis of the facet ligament and cartilage tissues at day 21. Capsular ligament collagen fibers stained with a Picrosirius Red/Alcian Blue stain^36^ were analyzed for the degree to which their orientation is random or aligned^37^, and such analysis reveals no difference (p = 0.731) in organization of the capsule’s collagen fibers between collagenase-and vehicle-injected joints (Fig. 2). Both groups exhibit a similar mean anisotropy index showing a mild level of alignment (mean ± SD; collagenase 0.45 ± 0.20; vehicle 0.45 ± 0.17) on a scale from 0 (isotropic, random) to 1 (aligned)^37^ (Fig. 2). Similarly, Mankin scoring quantification of Safranin O/Fast Green^36,38^ stained joints shows no differences (p = 0.444) in the structure and health of the bone and cartilage between groups (Fig. 3a), with a few of the collagenase-injected joints exhibiting very mild cartilage degeneration (Fig. 3a). Collagenase-injected joints exhibit a mean Mankin score of 2.54 ± 1.64 on a scale of 0 (healthy) to 10 (maximally degenerate)^38^ that is within one Mankin point of the vehicle group (3.45 ± 1.92), indicating no change in structure (Fig. 3a). In addition, neither joint space width (p = 0.841) nor cartilage width (p = 0.111) is altered by collagenase (Fig. 3a). Although chondrocytic HIF-1α expression increases with loading-induced temporomandibular joint pain^30^, the percentage of HIF-1α-positive cells in collagenase-injected joints (47.94 ± 24.72%) is not different from (p = 0.423) vehicle (53.31 ± 23.68%) (Fig. 3b), suggesting collagenase exposure that induces sensitivity (Fig. 1) is not sufficient to disrupt chondrocyte homeostasis at day 21.

### Intra-articular collagenase increases substance P and phosphorylated ERK expression in the DRG and spinal cord

**Figure 4:**
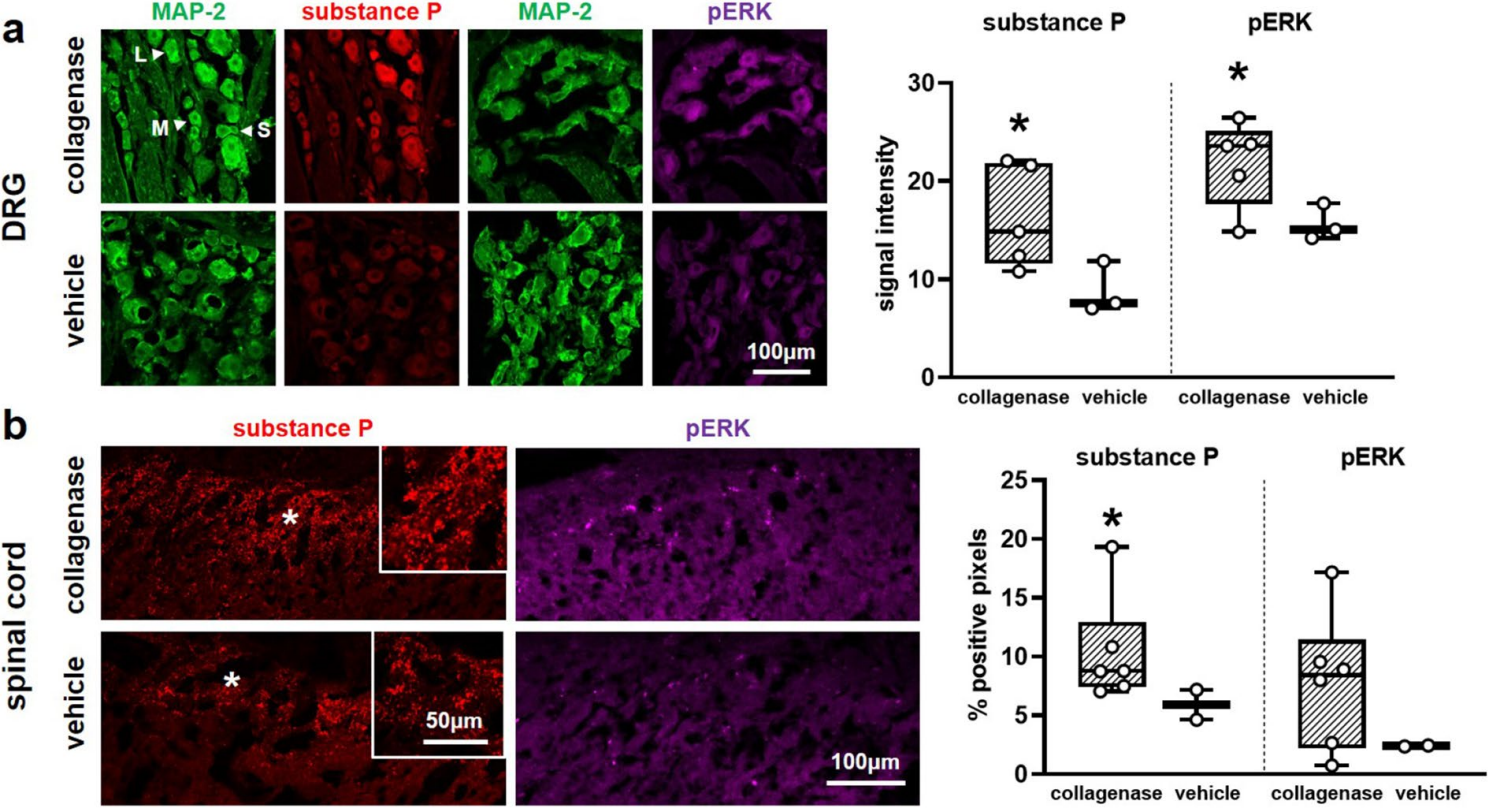
Immunolabeling of substance P and phosphorylated ERK in the dorsal root ganglia (DRG) and spinal cord at day 21. (**a**) Quantification of labeling intensity for the neurotransmitter substance P (red) and the MAP kinase pERK (purple) in cells with MAP-2 (green) positive labeling shows elevated expression after collagenase (5 rats) injection relative to vehicle (3 rats) injection for both substance P (Wilcoxon test; *p < 0.001) and pERK (Wilcoxon test; *p < 0.001) regardless of neuronal size (see Supplementary Fig. S1 online). White arrows indicate exemplary neurons identified as small (S), medium (M), or large (L) diameter neurons. Data points superimposed on box plots in (**a**) represent the mean for each rat (n = 5–7 images/rat; n = 10 cells/image). (**b**) Intra-articular collagenase (6 rats) also induces increased positive labeling for substance P (red) and pERK (purple) in the superficial dorsal horn. Spinal substance P (Wilcoxon test; *p = 0.039) is significantly greater after collagenase injection than after injection of the vehicle (2 rats), but the same increase is not detected with pERK expression (Wilcoxon test; p = 0.147). Spinal cord images show regions of the superficial dorsal horn (700 × 300 pixels) where immunolabels were quantified; insets show higher magnification of regions (white stars) to demonstrate positive substance P labeling. Individual data points on boxplots in (**b**) represent the mean value per rat quantified for 6–8 images/rat. For both panels, box-and-whisker plots show horizontal lines representing the first (lower) quartile, median, and third (upper) quartile of the data and whiskers represent the minimum and maximum values of the data set.

Immunolabeling of substance P and pERK in the DRG and spinal cord on day 21 suggests that collagenase may activate neuronal nociceptor and injury pathways in the peripheral and central nervous systems (Fig. 4). Expression of both substance P and pERK in MAP-2-positive DRG cells significantly increases (p < 0.001) in samples from collagenase-injected joints (Fig. 4a). Further, this effect is seen in neurons of all sizes; collagenase elevates substance P and pERK in small, medium, and large-diameter neurons (see Supplementary Fig. S1 online). Peripheral substance P levels after collagenase (14.95 ± 8.85) are nearly twice those of control (7.66 ± 4.94) and pERK levels increase by approximately 1.4-fold (collagenase 21.42 ± 9.65; vehicle 15.37 ± 6.10) (Fig. 4a). Positive labeling for both proteins is also evident in the superficial spinal dorsal horn (Fig. 4b). Although spinal substance P and pERK increase after intra-articular collagenase over levels for intra-articular vehicle, the difference is only significant for substance P (p = 0.039) (Fig. 4b).

### Increased peripheral and central MMP-1 expression coincides with dysregulation of nociceptive mediators

**Figure 5:**
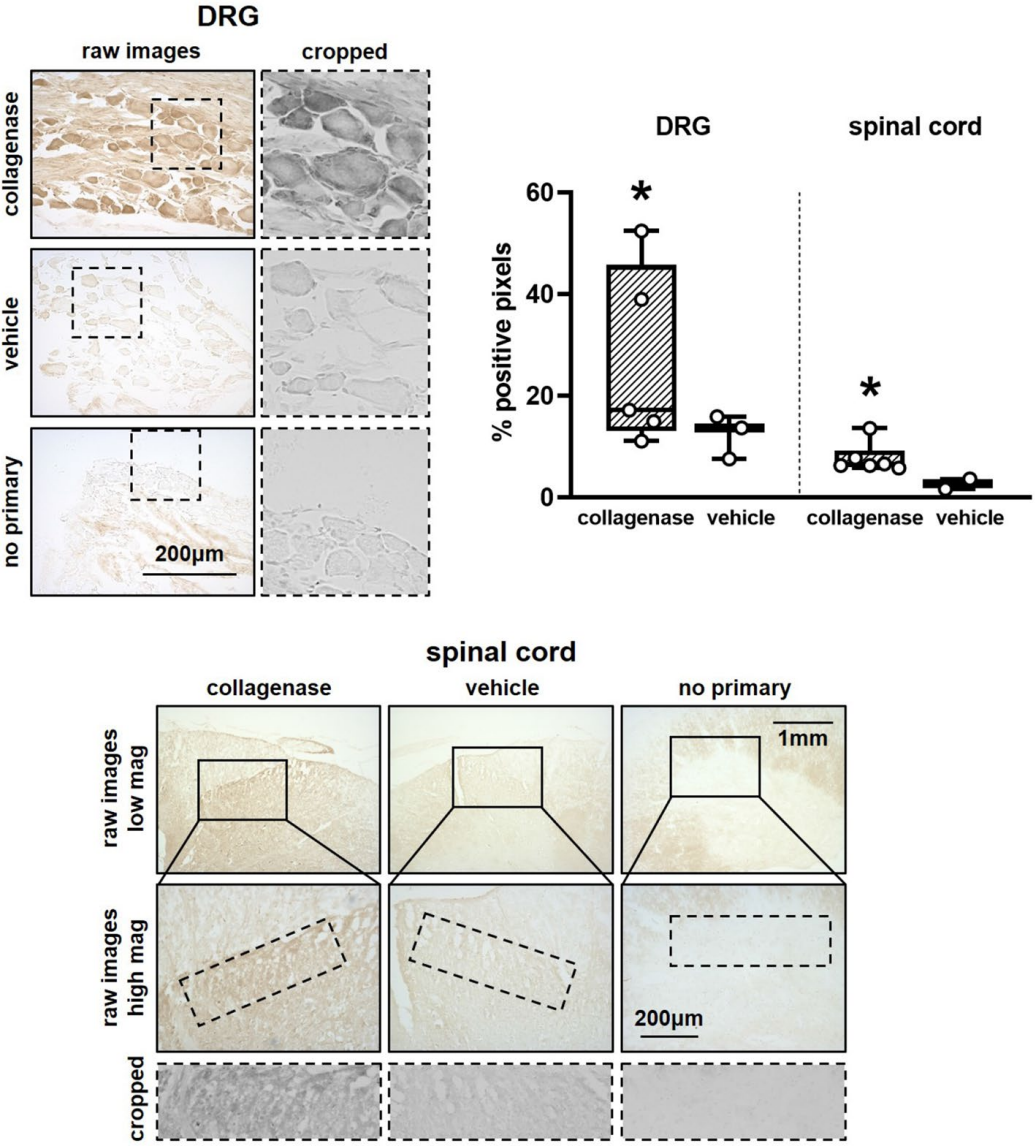
Detection of matrix metalloproteinase 1 (MMP-1) expression in the dorsal root ganglia (DRG) and spinal cord at day 21. Collagenase injection in the facet increases MMP-1 protein expression in the DRG (collagenase n = 5; vehicle n = 3; Wilcoxon test; *p = 0.039) with pronounced labeling in DRG cells. Dashed insets in the DRG images show representative cropped regions (600 × 600 pixels) where MMP-1 labeling is quantified in DRG cells. Spinal MMP-1 in the superficial dorsal horn is also greater with intra-articular collagenase (collagenase n = 6; vehicle n = 2; Wilcoxon test; *p = 0.041). Low magnification images (top) show the superficial spinal cord, from which high magnification images (middle) were acquired for quantification. Dashed insets in high magnification images show representative cropped (1500 × 500 pixels) regions where MMP-1 labeling is quantified in the superficial dorsal horn. Images of the no primary, negative controls are shown for both DRG and spinal cord labels. Box-and-whisker plots show horizontal lines representing the first (lower) quartile, median, and third (upper) quartile of the data. Data points for individual rats on boxplots represent the mean of 6–8 images/rat for each of the DRG and spinal cord quantification.

Given the mounting evidence for a role of MMP-1 in joint pain^16,39^ and our recent finding that neuronal MMP-1 increases in parallel with substance P in injured DRG neurons^10^, we also evaluated whether collagenase-induced pain-like behavior (Fig. 1) and elevated substance P expression (Fig. 4) are paralleled by changes in MMP-1 expression in the DRG and/or spinal cord. Indeed, MMP-1 expression increases both peripherally and spinally after intra-articular collagenase injection (Fig. 5). The average level of MMP-1 labeling in the DRG neurons of collagenase-injected rats (24.75 ± 22.48%) is significantly greater (p = 0.039) at twice that expressed in control rats with vehicle injections (12.50 ± 9.69%) (Fig. 5). Similarly, there is a significant increase in MMP-1 expression in the superficial dorsal horn (p = 0.041) of those rats receiving intra-articular collagenase (Fig. 5).

### A collagenase-induced loss of collagen alters neuronal activity and MMP-1 expression in an in vitro co-culture ligament model

Injecting crude bacterial collagenase in the facet joint and knee leads to degradative changes in the bone and cartilage of those joints and pain-like behaviors, in part, are attributed to such joint damage^24–26^. Yet, our in vivo results show that intra-articular collagenase induces behavioral sensitivity (Fig. 1) and neuronal dysregulation (Figs. 4, 5) without any evidence of substantial joint damage (Figs. 2, 3). We hypothesize that the same severe joint damage is not detected in our model because we use purified bacterial collagenase, which differs from the crude collagenase used in prior work^24–26^. The combination of proteolytic enzymes in crude collagenase catabolize other ECM molecules in addition to collagen, and in doing so, induce severe joint degeneration^40^. In contrast, the purified formula consists of two proteases with collagenolytic activity toward the alpha helices that comprise Type I, II, and III collagen and thus act *only* on collagen molecules^14^. Since bacterial collagenase does not act *directly* on afferent fibers, FLS cells, or other cellular populations in joint tissues, we hypothesize that purified collagenase induces sustained sensitivity (Fig. 1) and neuronal dysregulation (Figs. 4, 5) by acting on the collagen molecules in joint tissues. But, since tissue-level histological changes are not detected after collagenase (Figs. 2, 3), we posit that collagen molecule-level changes in vivo occur at a smaller scale than detectable by histology.

**Figure 6:**
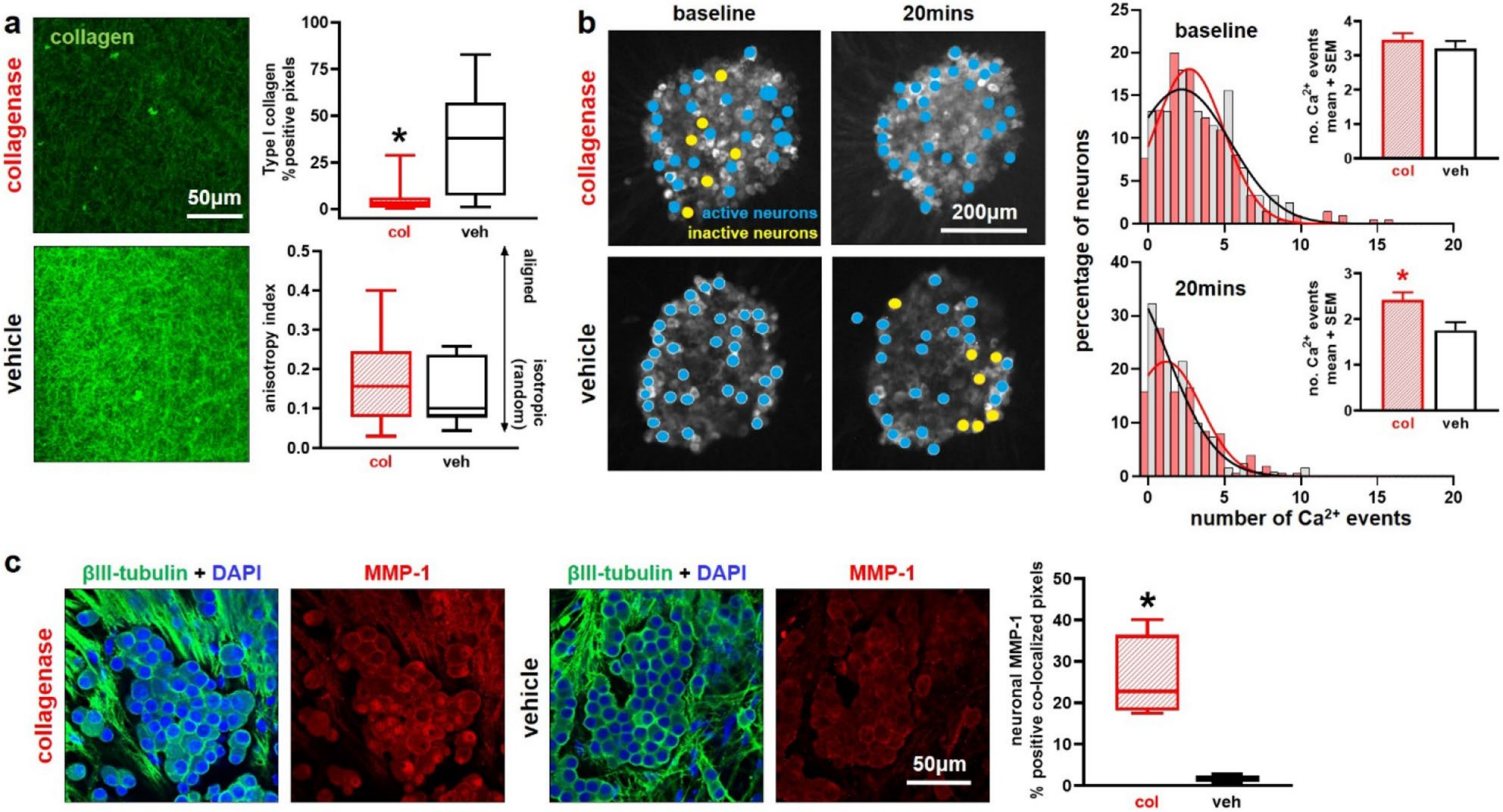
Effects of collagenase exposure on the collagen network and neuronal dysregulation in an in vitro co-culture model mimicking the capsular ligament. (**a**) Type I collagen labeling (green) after a 20 min collagenase (col) incubation (n = 4 gels/group; n = 4 images/gel) is significantly reduced from levels with vehicle (veh) exposure (Wilcoxon test; *p = 0.008); in contrast, fiber orientation is unchanged with collagenase exposure (two-tailed t-test; p = 0.380). (**b**) Time-lapse captures of fluorescent calcium imaging show DRGs with segmented individual neurons labeled as active (blue; ≥ 1 calcium events) or inactive (yellow; no calcium events) in images from both before the addition of either collagenase (7 DRGs) or vehicle (4 DRGs) solution and after 20 min of exposure. Neuronal firing profiles captured across 28–33 neurons/DRG are the same in both groups at baseline (Wilcoxon test; p = 0.562), shown by frequency distributions of the percentage of neurons versus the firing magnitude per minute. Summary data in the bar plot also show no difference in number of calcium events at baseline. However, firing frequency patterns differ with treatment after 20 min, with vehicle-exposed neurons quieting as shown by a left-shift of the frequency curve peak toward zero and the summary data detailed in the bar plot (Wilcoxon test; *vs. vehicle p = 0.002). (**c**) Collagenase exposure increases neuronal MMP-1 expression (collagenase n = 4 gels; vehicle n = 2 gels; Wilcoxon test; *p < 0.001) quantified by the co-localization of the neuronal structural protein βIII-tubulin (green) and MMP-1 (red); a DAPI label for nuclei is also shown (blue). Images show the maximum projection image of acquired confocal stacks (n = 5/gel) in regions with DRG axons and/or soma. Box-and-whisker plots in (**a**) and (**c**) show horizontal lines representing the first (lower) quartile, median, and third (upper) quartile of the data; whiskers are the minimum and maximum.

To test whether purified collagenase changes the collagen microstructure and initiates neuronal dysregulation we used our in vitro co-culture collagen gel model of the capsular ligament^10^ to measure higher-resolution cell and fiber responses in a system that preserves the cellular and structural microenvironment of the innervated joint capsule with less variability and complexity than in vivo. Co-cultures containing DRG neurons and FLS cells embedded in a Type I collagen gel that models the capsular ligament^10^ were incubated for 20 min in the same dose (60U) of purified bacterial collagenase as was used for the intra-articular injections. This incubation significantly decreases collagen labeling (p = 0.008), with collagenase-incubated gels (5.76 ± 7.72%) exhibiting a six-fold decrease relative to gels held in a control solution (36.28 ± 26.61%), suggesting substantial Type I collagen enzyme degradation during the 20 min incubation (Fig. 6a). Despite decreased collagen labeling, the organization of the fiber network is not changed by collagenase (p = 0.380) (Fig. 6a). Since collagen gels were fabricated with a random isotropic orientation, this finding suggests collagenase degrades fibers without preference to fiber orientation. A lack of effect of collagenase on fiber orientation in vitro is consistent with the observation that the collagen orientation in the rat’s capsular ligament is unchanged by intra-articular collagenase (Fig. 2).

To measure if neuronal function is also changed by exposure to collagenase, we assessed the physiological response of DRG neurons in those same co-cultures by measuring intracellular calcium influx as a proxy for action potentials^41^. In order to measure intracellular calcium, neurons were transduced with the adeno-associated virus and genetically engineered calcium indicator GCaMP6f under control of the Synapsin promoter that exclusively transduces neurons^41,42^. GCaMP6f fluoresces rapidly with the influx of calcium whereby neuronal fluorescence follows variations in intracellular calcium that occur with action potentials^41^. Calcium fluorescence traces in neurons were compared to a template-matching algorithm to identify calcium events indicative of action potentials^42^. The pattern of neurons with firing profiles before any exposure is not different (p = 0.562) (Fig. 6b), indicating that the neuronal populations exhibit equivalent activity and any change in firing patterns post-exposure is due to the exposure itself. After 20 min, neuronal firing profiles differ by treatment, with the distribution of vehicle-treated neurons exhibiting a greater number of inactive neurons and significantly fewer calcium events compared to those with collagenase exposure (p = 0.002) (Fig. 6b). The decrease in calcium events detected implies that those neurons quiet over time, whereas neurons exposed to collagenase maintain a heightened level of activity (Fig. 6b).

We also measured neuronal MMP-1 expression at that same time after incubation in order to assess if the collagenase-induced increase in MMP-1 in vivo (Fig. 5) is maintained in the co-culture model in association with sustained heightened neuron activity. Indeed, collagenase exposure significantly increases (p < 0.001) neuronal MMP-1 by nearly 15-fold (25.75 ± 10.10%) over that measured in co-cultures exposed to vehicle (1.74 ± 1.52%) (Fig. 6c). Analyses primarily focused on DRG neurons (Fig. 6c) to gain insight into if, and how, MMP-1 localizes to neurons since it is known to interact with neuronal cell-surface receptors^43–45^ and to parallel increases in neuronal substance P expression^10^. However, since fibroblasts also secrete MMP-1 and regulate its localization and sequestration in the multicellular space^8,46,47^, MMP-1 expression in FLS cells was analyzed and reveals that fibroblast-localized MMP-1 increases by nearly six-fold (collagenase: 31.93 ± 21.27%; vehicle: 5.40 ± 6.34%) after exposure to bacterial collagenase (p < 0.001; see Supplementary Fig. S2 online).

## Discussion

This study demonstrates that an intra-articular collagenase injection in the cervical facet joint alone is enough to induce sustained mechanical hyperalgesia and dysregulation of neuronal mediators in the periphery and spinal cord (Figs. 1, 4, 5). The increase in known nociceptive and afferent regulators (substance P, pERK) as well as the catabolic and signaling protease MMP-1 in DRG neurons (Figs. 4, 5) adds to growing evidence that trauma- and osteoarthritis-induced joint sensitivity is regulated by dysregulation of a host of neurotransmitters, cell signaling proteins, and matrix-altering proteins in the DRG^3,29,48,49^. Further, this occurs absent joint space narrowing, cartilage lesions, osseous changes, or chondrocyte disruption, all of which are pronounced in loading-induced osteoarthritis^30,31^ and with intra-articular crude collagenase^24–26^. Collagenase-induced changes in neural and joint outcomes (Figs. 2–5) occur coincident with mechanical hyperalgesia (Fig. 1), which is a proxy for peripheral sensitization; since this measurement is mechanically evoked, it does not fully capture the affective components of pain^3^. Integrating techniques such as the facial grimace scale in rats^50^ into in vivo models of joint pain would provide a measurement of spontaneous pain that is more translatable to the affective components of the pain experience in the human.

Our results imply that joint-mediated behavioral sensitivity from purified collagenase may develop and persist (Fig. 1) by different mechanisms than those etiologies with considerable joint degeneration. For example, contrary to our finding of elevated substance P in the DRG (Fig. 4a), crude collagenase in the knee decreases substance P expression in DRG neurons at 4 and 6 weeks post-injection despite the presence of pain-like behaviors^51^. The authors of that study infer that the decrease in substance P is due to afferents being injured by the severe articular destruction and subsequent exposure of subchondral bone caused by crude collagenase^51^. The opposite findings that substance P *decreases* with severe damage^51^ but *increases* without damage in our study (Figs. 2–4) are aligned with the disparity of observations related to structural destruction observed between the use of crude or purified collagenase^24–26^. The lack of change in chondrocytic HIF-1α (Fig. 3b) also differs from osteoarthritis models reporting HIFs are elevated with pain-like behaviors^30,31^. It is possible that HIF-1α may play a role in an earlier, yet transient, chondrocyte response to the intra-articular collagenase^31^. Yet, the lack of other evidence of cartilage damage (Fig. 3a) does not necessarily support this hypothesis and further highlights that pain-like behavior persists here absent the hallmark cartilage breakdown.

Notwithstanding the lack of tissue-level damage with collagenase-induced hyperalgesia (Figs. 1, 2, 3), afferent regulators can act peripherally to transmit pain within apparently undamaged joint tissues. The increase in substance P and pERK in the DRG (Fig. 4a) implies that collagenase in the joint induces synthesis of these, and likely other, proteins in neurons and would be available for distribution to peripheral synaptic terminals in innervated tissues. Moreover, pilot studies demonstrate that collagenase increases substance P and pERK expression in DRG monolayer culture^29^, absent a collagen substrate, suggesting that collagenase may increase these proteins independent of its influence on collagen molecules. Although we did not probe substance P in joint tissues, its expression has been shown to increase in subchondral bone several weeks after administration of crude intra-articular collagenase^25^, suggesting substance P could also be transported peripherally in our model and contribute to peripheral sensitization (Fig. 1). The increased phosphorylation of ERK in the DRG that is evident in our model (Fig. 4a) supports the notion of peripheral sensitization since ERK is activated in neurons in response to intense noxious stimulation^32^.

Our finding that collagenase increases pERK and substance P expression in neurons across all sizes (Fig. 4a; Supplementary Fig. S1 online) suggests that collagenase affects several neuronal populations that are functionally and molecularly heterogeneous^12^. In their most common characterization during non-pathologic states, small-diameter neurons are nociceptors that transmit “slow pain” via unmyelinated C-fibers, medium-diameter neurons are nociceptors that transmit “fast pain” via Aδ myelinated fibers, and large-diameter neurons are mechanoreceptors that relay touch sensation via Aβ myelinated fibers^12^. Since ERK phosphorylation occurs within minutes of a noxious stimulus in small- and medium-diameter neurons^32,52^, its elevation at the late time point of day 21 after collagenase (Fig. 4a; Supplementary Fig. S1), but when behavioral sensitivity is still present (Fig. 1), suggests there may be ongoing noxious input from the joint. Of note, peripheral noxious input does not appear to involve injury to the joint’s chondrocytes since HIF1α levels are unaltered (Fig. 3b). The phosphorylation of ERK in large-diameter neurons indicates that some degree of tissue injury may be occuring after collagenase injection (Supplementary Fig. S1), since ERK is only phosphorylated in mechanoreceptors after nerve injury^53^. This widespread ERK phosphorylation across DRG neurons likely contributes to the development of behavioral sensitivity (Fig. 1).

Collagenase also increases substance P in neurons of all sizes, with the highest intensity of labeling in small- and medium-diameter nociceptors (see Supplementary Fig. S1 online), suggesting that the behavioral sensitivity caused by intra-articular collagenase (Fig. 1) may be transmitted via both the primary and secondary populations of nociceptors. Although this analysis does not distinguish between the peptidergic and nonpeptidergic neurons that make up the small-diameter, C-fiber population, the small-diameter neurons where substance P increases are likely the peptidergic population that release neuropeptides like substance P and calcitonin-gene related peptide (Fig. 4a; Supplementary Fig. S1)^12^. Furthermore, the hyperalgesia we report in the forepaw is secondary hyperalgesia^12^ since it is remote from the C6/C7 injection site. It is possible that abnormal recruitment of mechanoreceptors in nociception contributes to the manifestation of hyperalgesia away from the injury site^12^, since substance P increases in large-diameter neurons. Of note, the percentage of neurons positive for protein expression was not assessed, instead, we used a semi-quantitative technique to determine the level of expression^54,55^. Further, although collagenase exposure results in a greater number of calcium events than vehicle (Fig. 6b), this effect is measured across all neurons, as the Synapsin promoter used here does not selectively transduce neurons by type^41,56^. Further refining assessment of the population of neurons in which afferent regulators increase could lend deeper insight into how collagenase leads to noxious transmission and the molecular receptors involved.

Central sensitization may also contribute to the manifestation of behavioral sensitivity from elevated spinal substance P (Figs. 1, 4b), but to a different extent than peripheral sensitization given that spinal pERK levels are not elevated (Fig. 4b). Increased substance P in the superficial dorsal horn (Fig. 4b), where peptidergic C and Aδ afferent fibers synapse, suggests that collagenase may induce central sensitization via enhancement of nociceptive circuits decoupled from peripheral noxious input^12,57^. Indeed, there is evidence of central pain processing after joint trauma^58^, in experimental osteoarthritis models^59^, and in patients with osteoarthritis knee pain who exhibit signs of central sensitization using quantitative sensory testing^60,61^. Although widespread, systemic sensitivity is evident in patients with osteoarthritis^60^, we did not measure whether sites further from the forepaw exhibit sensitivity in this study. Despite evidence for central sensitization, the ability of clinical interventions such as local intra-articular anesthetics and total joint replacement to alleviate joint pain suggests that ongoing peripheral input from the affected joint, at least in part, may drive joint pain at chronic stages^62^. In fact, the relative contributions of central and peripheral sensitization have been proposed to vary by patient and may explain the disagreement between pain severity and radiographic joint damage whereby patients with little damage exhibit higher physiological evidence of central sensitization, and vice versa^60^. Although substance P signaling contributes to neuronal hyperexcitability^12,57^, electrophysiology data from DRG and spinal neurons are needed to identify if, where, and when, aberrant firing occurs. The ability of collagenase to maintain firing profiles in live neurons (Fig. 6b) supports that collagenase influences neuronal signaling at least very early after exposure, but neuronal protein assays represent a snapshot in time (at day 21 in this case), and do not inform about the temporal development of neuronal hypersensitivity.

The collagenase used in this study is from the bacterium *Clostridium histolyticum*, and is a foreign body except during certain bacterial infections^14^. So, it is devoid of the many other pathophysiological roles of native MMPs^13,63^ and effectively isolates *only* the collagen catabolism function of native collagenases. As such, intra-articular collagenase is not taken as a model of osteoarthritis, but rather purified bacterial collagenase enables answering questions about the role of collagen catabolism in degenerative joint diseases. Because of this “simple” effect, collagenase presumably initiates nociceptive cascades via a microscale degradation of collagenous tissues in the joint and not through non-collagenolytic-dependent pathways such as direct receptor binding on neurons^44,45,64^. It should be noted that some of the injected collagenase leaves the joint space immediately, and the remainder of the enzyme is likely cleared within several hours^18^; yet, neither the volume or clearance of injectant were measured in this study. Despite the likelihood of rapid clearance from the joint space^18^, the decrease in Type I collagen labeling observed in the co-culture collagen gels supports that collagen is digested robustly even within minutes (Fig. 6a). Yet, that collagenase exposure does *not* change the organization of fiber orientation in either the capsular ligament (Fig. 2) or in the co-culture collagen gels (Fig. 6a). Certainly, a lack of orientation differences does not rule out that the degradation that is observed in the in vitro ligament model may also be occurring in the capsular ligament as well. Taken together with the lack of overt changes collectively in either the ligament or cartilage (Figs. 2, 3), we propose that even very subtle collagen changes induced by enzyme-mediated degradation are enough to cause long-lasting behavioral sensitivity and that these changes may not be readily apparent on radiographs. Notably, this study used only a single dose and did not investigate whether the behavioral sensitivity, joint, and/or neural outcomes vary with the amount of bacterial collagenase. Investigating whether lower and/or more frequent collagenase doses induce transient and/or sustained behavioral sensitivity would provide insight into how “tunable” collagen catabolism affects manifest pain-like behaviors.

Collagenase-mediated collagen breakdown could initiate nociception by altering the microstructure of the collagen network surrounding afferents in the facet capsule leading to aberrant cell–matrix interactions. Indeed, prior studies in knockout mice implicate Type IX and Type V collagen directly in the manifestation of pain-related behaviors^65,66^. Mechanosensitive afferent fibers and FLS cells embedded in the collagenous ligament respond to changes in their mechanical microenvironment via adhesive interactions with the ECM^7^; such disruption of collagen types involved in ECM cohesion may directly induce several behavioral characteristics of pain^66^. Moreover, mechanically-triggered neuronal^67^ and fibroblast^46^ responses depend on collagen concentration , so a collagenase-induced decrease in collagen (Fig. 6a) could alter how cells respond to mechanical cues after exposure. Collagen breakdown could interfere with transmembrane integrin receptors that mediate collagen-axon adhesions, for example, and regulate substance P-mediated signaling^7^. In fact, a collagenase-induced reduction in collagen by nearly 50% compromises the tensile biomechanical responses of gels using the same formulation as in the current study^29^. The collagenase exposure used here decreases collagen labeling by approximately 87% (Fig. 6a), so it is likely that if subjected to load, degraded co-cultures would be weaker, less stiff, and reorganize differently, and as such, alter cell–matrix interactions.

Although it is well-established that nociceptive signaling is activated in afferents by *stretch-induced* changes to their local matrix^7,11,67^, this study suggests that *degradation-induced* changes alone, absent any stretch, may also affect neuronal signaling. This notion is supported by the maintained neuronal signaling (Fig. 6b) and elevated neuronal MMP-1 expression (Fig. 6c) after collagenase digestion absent any additional mechanical stimulus. The higher number of calcium events in collagenase-exposed neurons (Fig. 6b) suggests a possible mechanism by which degradation-induced changes to the collagen network may regulate neuronal firing; although this requires further investigation and electrophysiological data to fully understand. It is also possible that the degradation itself does not initiate nociception, but rather weakens the biomechanics of the collagen matrix and lowers the neuronal threshold for mechanical activation. In fact, exposing isolated rat facet joints to the same collagenase digestion also lowers the failure force and stiffness of the capsular ligament^68^. There is also more abnormal microstructural reorganization of fibers in collagenase-treated capsules at strains associated with pERK upregulation^11,68^, further supporting that stretch of a degraded ligament can initiate injury and nociceptive signals in neurons, transmitting pain from the periphery. This notion is further supported by studies of human classical Ehlers-Danlos syndrome in mice, whereby Type V collagen deficiency compromises joint stability and causes generalized sensitization^65^. A reduced threshold for mechanical activation of nociceptive afferents in degraded capsules could explain the hyperalgesia detected with collagenase (Fig. 1), as well as pain symptoms experienced by patients with osteoarthritis during usually non-injurious movements like walking or climbing stairs^3,20^.

It is also possible that collagenase produces collagen degradation products that act as signaling peptides in the joint. Collagen fragments produced by MMP-degradation are detectable in synovial fluid, serum, urine, and plasma in patients with painful joint degeneration^69^ and can act as ligands in cell–cell signaling^70^. Although the vast majority of studies characterizing collagen degradation products in humans have measured products of cartilage degradation and bone resorption, like CTX-II and COMP^69^, a few show that the Type I collagen degradation fragment C1M is detectable in serum of patients with symptomatic osteoarthritis^71–73^. C1M levels and hypersensitivity trend towards a positive correlation^71^, suggesting a clinically-relevant relationship between Type I collagen degradation and nociception. Although the initial mechanism of cleavage differs between native interstitial collagenases (the human MMPs) and bacterial collagenases, both kinds of enzymes produce collagen fragments that can be subsequently post-processed into fragments < 40 kDa^14,18,74^; those fragments are measurable in the synovial fluid within minutes of an MMP-13 intra-articular injection in the hamster^18^. Small collagen fragments ranging between 2.7–15.6 kDa bind directly to the α2A domain of integrin^70^. Since neurons express integrin receptors and integrin-signaling is involved in the transduction of noxious stimuli^7,75^, it is possible that small collagen fragments may also bind to neuronal receptors, or indirectly influence neuronal signaling through their regulation of integrin. Such collagen fragment-mediated cascades could play a role in the development of behavioral sensitivity (Fig. 1) and neuronal dysregulation (Fig. 4, 5) observed in our model. Although we show an unequivocal decrease in collagen in vitro (Fig. 6a), the degradation of collagen is not quantified in the rat study. Measuring Type I collagen degradation products like C1M and its interactions with neurons is a promising direction for future work.

We have previously established relationships between neuronal substance P and MMP-1 expression in our co-culture model of the capsular ligament after injurious stretch^10^. The increase of both proteins in DRG neurons and the spinal cord in this study suggests MMP-1 and substance P expression could be related in collagenase-induced behavioral sensitivity (Figs. 1, 4, 5). Furthermore, the collagenase-induced increase in neuronal MMP-1 in vitro (Fig. 6c) corroborates a possible causal relationship between intra-articular collagenase and upregulation of MMP-1 (Fig. 5). In normal non-pathologic tissues, MMP-1 levels are usually low^63^, so the fact that MMP-1 is detectable and elevated over controls in the rat DRG and in co-cultures (Figs. 5, 6c; Supplementary Fig. S2 online) indicates a distressed cellular state. MMPs are regulated in part by fibroblasts and interactions with ECM components, including matrix turnover^13,46,47^, so degradation of collagen (Fig. 6a) may be responsible for its elevation (Figs. 5, 6c; Supplementary Fig. S2 online). As such, an increase in fibroblast-localized MMP-1 (see Supplementary Fig. S2 online), at least in joint tissues where degradation is presumably localized, would be expected; yet, MMP-1 localization to neurons in both co-culture gels (Fig. 6c) and DRG tissue (Fig. 5) is surprising. MMP-1 localization to neurons may directly stimulate action potentials and trigger abnormal firing patterns since there are known relationships between MMP-1 and cell surface receptors and non-matrix substrates involved with nociception^13,44,45,64^. Exogenous MMP-1 increases neuronal excitability^43^, so its elevation in co-cultures and its localization to neurons may maintain neuronal signaling (Fig. 6) ; it also suggests MMP-1 may contribute to peripheral and/or central hyperexcitability in the rat (Fig. 5).

Overall, these studies put forth a model of how nociception is derived from an apparently structurally unaffected joint, effectively mimicking the clinical scenario in which symptomatic joint pain patients do not present with hallmark evidence of joint destruction on imaging^20,22,23,60^. We speculate that microscale collagen degradation leads to behavioral sensitivity and some combination of peripheral and/or central alterations to nociceptive processing via altered cell–matrix interactions or production of potent collagen fragment ligands. Our data support the emergent belief that changes in the composition of joint tissues, such as a loss of Type I collagen, are an early initiator of degenerative joint disease that precedes “currently detectable” pathology^20^. Still, there remain many challenges in detecting microscale collagen damage clinically. Advances in MRI and other imaging modalities are beginning to achieve greater sensitivity than the characteristic radiographs used for a clinical joint degeneration diagnosis. MRI features, like bone marrow lesions, synovitis, and effusion, show promise as stronger correlates with pain^20^, and support that pain without overt joint destruction could be due to inflammatory changes in joint tissues in concert with, or instead of, microscale collagen degradation^71,76–78^. The ability of nonsteroidal anti-inflammatory drugs to attenuate pain-related behaviors after intra-articular collagenase^25,26^, as well as pilot studies showing increased gene expression of cytokines with collagenase in vivo and in vitro^79,80^, support that inflammation may play a role in the manifestation of hyperalgesia (Fig. 1). Even so, the associations with inflammatory MRI features and pain are still inconsistent and MRI “abnormalities” are common in otherwise painless joints^20^. As substance P, pERK, and MMP-1 appear to be indicators of microscale Type I collagen degradation, these molecular regulators should be considered in diagnostic and therapeutic advances as facilitators of hyperalgesia in joint diseases that involve pathologic Type I collagen degradation.

## Methods

### Animals

All procedures were approved by the University of Pennsylvania IACUC and performed under the Committee for Research and Ethical Issues of the IASP guidelines^81^. Male Holtzman rats (Envigo; Indianapolis, IN; 476 ± 41 g at termination) were pair-housed with 12-h light/dark cycles and randomly assigned to groups for injection (collagenase n = 12; vehicle n = 6). After behavioral testing on day 21, cervical spinal facet joints (collagenase n = 6; vehicle: n = 3) or neural tissues (collagenase n = 6; vehicle: n = 3) were harvested from separate rats. Groups were evaluated simultaneously; all quantitative analyses were performed without group identification to eliminate bias. DRGs from all spinal levels were taken from embryonic day 18 Sprague–Dawley rats obtained from the CNS Cell Culture Service Center of the Mahoney Institute of Neuroscience for co-cultures. Embryonic DRGs were harvested according to prior methods and to ensure a high content of DRGs while minimizing the required animals^82,83^. FLS were harvested from hind knees of a sexually mature adult male Holtzman rat (384 g).

### Intra-articular injection & tissue harvest

Under inhalation isoflurane anesthesia (4% induction; 2.5% maintenance), a midline incision over the back of the neck exposed the paraspinal musculature of the C4–T2 vertebrae. The C6/C7 facet joints were cleared, and injected bilaterally with either purified bacterial collagenase (collagenase n = 12) dissolved in saline (10 μL; 60 U; CLSPANK; Worthington Biochemical Corporation; Lakewood, NJ) or saline (10μL; vehicle n = 6). Wounds were sutured and stapled; rats were recovered in room air. Surgical staples were removed after 14 days, and weight gain was continuously monitored.

On day 21, rats were anesthetized with sodium pentobarbital (65 mg/kg; i.p.) and transcardially perfused with phosphate-buffered saline (PBS; 250 ml) followed by 4% paraformaldehyde (PFA; 250 ml). The C4-T1 spinal columns were harvested from half of the cohort (collagenase n = 6; vehicle n = 3) and post-fixed in 4% PFA for 24hrs, held in sucrose (Sigma; St. Louis, MO) dissolved in PBS (30%) for 7 days, and decalcified in 10% EDTA (Thermo Fisher; Waltham, MA) for 3–4 weeks^34^. The C6/C7 segment was embedded in Tissue-Tek OCT Compound (Fisher Scientific; Waltham, MA), coronally cryosectioned (16 μm), and thaw-mounted onto Superfrost Plus slides (Fisher Scientific). DRGs and spinal cord at C6/C7 were harvested from the remaining rats (collagenase n = 6; vehicle n = 3), post-fixed in PFA for 24 h, held in 30% sucrose for 7 days, and embedded in OCT. Axial cryosections (14 μm; 6–8/rat) were thaw-mounted. Tissue sections from naïve rats (n = 2) were included in all analyses to provide un-operated control tissue.

### Behavioral testing

Forepaw mechanical PWTs were measured bilaterally to quantify mechanical hyperalgesia using customary methods^34^. Von Frey filaments of ascending strengths (Stoelting; Wood Dale, IL) were applied to each rat’s forepaw and responses identified using thresholding methods^34^. PWTs were quantified before surgery (baseline; BL) and on days 1, 3, 5, 7, 11, 14, 17, and 21. Three rounds of testing were completed each day, separated by 10mins; all rounds were averaged bilaterally to obtain the daily PWT for each rat.

### Picrosirius Red/Alcian Blue staining

Tissue sections were stained with Picrosirius Red/Alcian Blue^36^ (Sigma) to visualize collagen fibers in the capsular ligament, and imaged using an EVOS FL Auto Imaging microscope with a 20 × Olympus objective (5–6 images/rat). Separate regions of interest (ROIs) containing only the ligament were randomly selected (2–4 ROIs/image) and fiber orientation was analyzed using a Fourier transform method, which computes the magnitude and direction of the principal orientation axes of the image^37^. The anisotropy index was calculated from the ratio of the principal axes to describe orientation on a continuous scale from isotropic (random; 0) to aligned (1)^37^ and averaged across ROIs for each rat.

### Safranin O/Fast Green staining

Cartilage structure was evaluated using Safranin O/Fast Green staining (Sigma) to visualize cartilage and bone^36^. Joint sections were prepared and imaged as described for ligament. Stained articular surfaces (6–8 images/rat) were scored by two blinded graders using a modified Mankin scale to assess cartilage degradation^38^, based on cellular and background staining, chondrocyte arrangement, and structural surface condition, with scores ranging from normal (0) to maximally degenerate (10)^38^, and averaged across all images for each rat.

Joint space and cartilage width were measured using FIJI (NIH; Bethesda, MD) to evaluate joint space narrowing and cartilage degradation^5^. Joint space width was quantified as the perpendicular distance between the articular surfaces; cartilage width was defined by the length of the Safranin O staining perpendicular to the subchondral bone (Fig. 3a). Both measurements were made in triplicate for each image (3–4 images/rat) from the lateral to medial end of the joint and averaged.

### HIF-1α immunolabeling

HIF-1α expression was probed using immunolabeling to assess the health of chondrocytes in C6/C7 articular cartilage^30,31^. Immunohistochemical protocols are described previously^31^ and used a primary antibody for rabbit anti-HIF-1α (1:250; Abcam Cambridge, MA) and a biotinylated horse anti-rabbit IgG secondary antibody (1:1000; Vector Laboratories; Burlingame, CA). Tissue sections without primary antibodies were included as negative controls and to verify antibody specificity for all immunolabeling assays. Articular cartilage was imaged using an EVOS FL Auto Imaging microscope with a 40 × Olympus objective. The acquired 40 × images (5 sections/rat) were cropped in FIJI to areas of 1000 × 450 pixels to include regions with chondrocytes (Fig. 3b). An assessor blinded to groups counted both the number of HIF-1α-positive and total number of cells in each image^31^; the percentage HIF-1α-positive cells was taken as an average for each animal.

### Immunolabeling of neural tissue for substance P, pERK & MMP-1

To assess substance P, pERK, and MMP-1 in the peripheral and central nervous systems, axial cryosections of C7 DRGs and spinal cord (6–8/rat) were immunolabeled. Neural tissue was triple-labeled for MAP-2 (chicken; 1:500; Abcam), substance P (guinea pig; 1:400; Neuromics; Edina, MN), and pERK (mouse; 1:500; Cell Signaling; Danvers, MT). Sections were triple washed in TBS (Thermo Fisher Scientific) with Triton X-100 (0.03%; Bio-Rad; Hercules, CA), blocked in TBS with normal goat serum (10%; Vector) and BSA (1%; Sigma) for 2 h at room temperature, and incubated overnight at 4 °C with primary antibodies in TBS with 1% BSA. Sections underwent three washes and were incubated for 2 h at room temperature with the Alexa Fluor secondary antibodies goat anti-chicken 488, anti-guinea pig 633, and anti-mouse 568 (all 1:1,000; Thermo Fisher) in TBS with BSA (1%). After washing in TBS and deionized water, slides were cover-slipped with Fluorogel (Electron Microscopy Sciences; Hatfield, MA).

Sections that were fluorescently-labeled for MAP-2, substance P, and pERK were imaged at 20 × with a Leica TCS SP8 confocal microscope. To quantify substance P and pERK labeling intensity in neurons, MAP-2 positive cells (n = 10/image) were first identified by a blinded grader (n = 5–7 images/rat) to select neurons (Fig. 4a); then, the signal intensity of substance P and pERK labeling in the MAP-2 selected neurons was separately quantified by manually outlining the neurons and quantifying the average pixel brightness, using FIJI (see Fig. S1 in Supplementary Information online). The diameter of each cell was also calculated as the average of the length and width of each selected region to analyze substance P and pERK immunolabels by neuronal size (see Fig. S1 in Supplementary Information online). To quantify substance P and pERK in the superficial dorsal horn where nociceptors synapse^12^, spinal cord images were cropped to include only the superficial dorsal horn which corresponds to an area of 700 × 300 pixels; substance P and pERK were quantified, separately, by counting the number of pixels above the threshold for expression in naïve tissue^48^. DRG signal intensities and the percentage of positive pixels in spinal cord were quantified for each image and averaged by rat.

To quantify MMP-1 in neural tissue, separate cryosections (6–8/rat) were washed in distilled water, incubated in Dako Target Retrieval Solution (Agilent; Santa Clara, CA) for 30mins at 95 °C, cooled, and processed as described for HIF-1α^31^ using a primary antibody for MMP-1 (rabbit; 1:400; Proteintech; Rosemont, IL). MMP-1 labeled tissues were imaged using an EVOS FL Auto Imaging microscope with a 40x (DRG) or 20x (spinal cord) Olympus objective. Areas of 600 × 600 pixels were cropped from the raw 40 × DRG images to exclude connective tissue (Fig. 5). Areas of 1500 × 500 pixels were cropped from the raw 20 × spinal cord images to include only the superficial dorsal horn (Fig. 5).MMP-1 expression was quantified using densitometry in the cropped 600 × 600 pixel DRG images and in the cropped 1500 × 500 pixel spinal cord images^48^. The percentage of positively labeled pixels was separately calculated in DRG and spinal cord and averaged by rat.

### In vitro co-cultures: fabrication, collagenase exposure, collagen assessments, neuronal calcium imaging & MMP-1 immunolabeling

Type I collagen gels (2 mg/mL) were fabricated with FLS (~ 5 × 10^4^ cells/mL) and DRGs (6–10/gel)^10^, and cultured until day-in-vitro (DIV) 10. On DIV10, co-culture gels were incubated with 60 U of purified bacterial collagenase (CLSPANK; Worthington) in DMEM (collagenase) or DMEM only (vehicle) for 20mins, since gel mechanics are altered by collagenase exposures for 15-35mins and 20mins allows enough time to detect neuronal changes^10,29,43^.

To assess the effect of collagenase on the amount of collagen and its organization, a subset of gels (collagenase n = 4; vehicle n = 4) was fixed (4% PFA) after 20mins of incubation at 37 °C and immunolabeled for Type I collagen using a free-floating protocol with normal goat serum (Vector), a primary antibody to collagen (mouse; 1:400; Abcam) and the Alexa Fluor secondary antibody goat anti-mouse 488 (1:1,000; Thermo Fisher)^7^. Image stacks (n = 4/gel) were acquired at 10 μm steps over a 50 μm depth at 40 × with a Leica TCS SP8 confocal microscope. A single maximum projection image from each stack was analyzed to quantify the number of positive pixels^10^. Collagen fiber orientations were analyzed using the Fourier transform method described for ligament analyses^37^.

To measure neuronal activity during collagenase exposure, DRG neurons were transduced on DIV2 by adding the adeno-associated virus expressing GCaMP6f (#AAV1.Syn.GCaMP6f.WPRE.SV40; 1:4000) in culture media. GCaMP6f rapidly and transiently fluoresces with calcium influx^41^ and enables the visualization of calcium transient waveforms in real-time. In a subset of gels incubated in collagenase (n = 5) or vehicle (n = 2), time-lapse images were acquired (20 Hz; 1 min) at baseline and after 20mins with a Nikon Eclipse TE2000U spinning disk confocal microscope (CSU-10b; Solamere Technologies; Salt Lake City, UT) in an environmental chamber held at 37 °C and 5% CO_2_^42^. For each gel, 1–2 DRGs were imaged, maintaining a constant field of view. The same DRGs were imaged at both baseline and 20mins so that time-lapse data tracked the same DRGs over time. Fluorescence data were analyzed using FluoroSNNAP in MATLAB (MathWorks)^42^. Individual neurons (n = 28–33) were segmented in each DRG; although neurons in the same DRG were segmented at both timepoints, the segmentation of identical neurons could not be maintained due to motion artifact caused by collagenase-induced degradation during the exposure. Only neurons with a cell nucleus distinct from the cytosol were selected to ensure that only living neurons were analyzed. The number of Ca^2+^ events was counted using a template-matching algorithm to identify Ca^2+^ waveforms that are known to match those that occur with action potentials^42,84^. The total Ca^2+^ events across all segmented neurons was calculated for each DRG and concatenated for each group at each timepoint.

Separate gels incubated in collagenase (n = 4) or vehicle (n = 2) were fixed (4% PFA) after 20 min of incubation at 37 °C to assess effects of collagenase on neuronal MMP-1 expression using immunolabeling. Gels were fluorescently labeled using the same protocol as above, absent BSA, and with primary antibodies to MMP-1 (rabbit; 1:200; Proteintech) and βIII tubulin (anti-mouse; 1:300; Biolegend; San Diego, CA). After incubation with the Alexa Fluor secondary antibodies goat anti-rabbit 555 and anti-mouse 488 (both 1:1000; Thermo Fisher), gels were incubated in DAPI (1:200; Thermo Fisher) at room temperature for 15 mins to stain cell nuclei. Im ages of DRG axons and somas (n = 5/gel) were acquired at 40 × with a Leica TCS SP8 confocal microscope. Labeling above a threshold for positive MMP-1 and βIII tubulin labeling was separately quantified^10^ and their co-localization computed and normalized to total βIII tubulin as a measure of neuronal MMP-1.

### Statistical analyses

All statistical analyses were performed with α = 0.05 using JMP Pro v14 (SAS Institute Inc.; Cary, NC). Normality was tested using a Shapiro–Wilk goodness-of-fit test for a normal continuous fit on the residuals of all outcomes. PWTs are expressed as mean ± standard deviation. Differences in PWT between groups over time were compared using repeated-measures ANOVA and post-hoc Tukey HSD tests, with a single rat as an experimental unit. Anisotropy index, joint space width, cartilage width, and HIF-1α expression were compared by two-tailed t-tests. Since the Mankin score and all immunolabeling outcomes had non-normal distributions (p < 0.05; Shapiro–Wilk test), differences were tested with a non-parametric Wilcoxon (Mann–Whitney) test.

Similarly, a Wilcoxon test assessed the differences in collagen labeling between groups. A t-test compared anisotropy index between gel exposures. Differences between the distribution of calcium events were tested with a Wilcoxon test, separately at each of baseline and 20 min. Neuronal MMP-1 labeling was compared using a Wilcoxon test since data were non-normally distributed.

## Supplementary Information

Supplementary Figures.

## Data Availability

Datasets generated and/or analyzed during this study are available from the corresponding author on reasonable request.
