# Supplementary material for: Intra-articular collagenase in the spinal facet joint induces pain, DRG neuron dysregulation and increased MMP-1 absent evidence of joint destruction
Source: Sci Rep. 2020 Dec 15;10:21965. doi: 10.1038/s41598-020-78811-3 (PMC7738551; doi:10.1038/s41598-020-78811-3)
Supplement: Supplementary file 1 — Supplementary Figures. [file 41598_2020_78811_MOESM1_ESM.docx]

**SUPPLEMENTARY INFORMATION**

**INTRA-ARTICULAR COLLAGENASE IN THE SPINAL FACET JOINT INDUCES PAIN, DRG NEURON DYSREGULATION AND INCREASED MMP-1 ABSENT EVIDENCE OF JOINT DESTRUCTION**

Meagan E. Ita, M.S.,^a^ Prabesh Ghimire, M.S.,^a^ Rachel L. Welch,^a^ Harrison R. Troche,^a^ Beth A. Winkelstein, Ph.D.^a,b*^

^a^ Department of Bioengineering, University of Pennsylvania, Philadelphia, PA 19104

^b^ Department of Neurosurgery, University of Pennsylvania, Philadelphia, PA 19104

^*^ winkelst@seas.upenn.edu

**CORRESPONDING AUTHOR:**

Beth A. Winkelstein, PhD

Dept. of Bioengineering

University of Pennsylvania

210 S. 33^rd^ Street

240 Skirkanich Hall

Philadelphia, PA 19104-6392

1-215-573-4589 (phone)

1-215-573-2071 (fax)

winkelst@seas.upenn.edu

**Co-Authors:**

Meagan E. Ita

meita@seas.upenn.edu

Prabesh Ghimire

prabesh@seas.upenn.edu

Rachel L. Welch

rachel.welch@yale.edu

Harrison R. Troche

hrtroche@gmail.com

**Immunolabel quantification & size classification in the DRG**

**Figure S1. Substance P and pERK quantification in the DRG by neuronal size.** The low magnification image in (a) shows a merged MAP-2 (green) and substance P (red) immunolabel with blown out insets of the separated MAP-2 and substance P channels. Substance P and pERK are quantified in the isolated confocal image channels by first identifying 10 randomly selected MAP-2 positive cells in the MAP-2 imaged channel (white asterisks) and then manually outlining those same neurons in either the substance P or pERK imaged channel using the FIJI software; the substance P channel is shown with selected neurons outlined in yellow. The average pixel intensity within, and the major and minor diameter of, the elliptical regions were then measured in the outlined neurons using FIJI to quantify signal intensity and neuronal size, respectively. (b) Intra-articular collagenase (col; 5 rats) increases substance P and pERK expression over intra-articular vehicle (veh; 3 rats) in small (<21μm), medium (21-40μm), and large (>40μm) diameter neurons (Wilcoxon tests; *p≤0.029). In the neurons assessed from the collagenase group (n=337 neurons), 14.3% are small, 60.9% are medium, and 24.8% are large; neuron proportions are similar in the vehicle group (n=220 neurons; 13.2% small, 61.8% medium, 25.0% large). Box-and-whisker plots show horizontal lines representing the first (lower) quartile, median, and third (upper) quartile of the data.

**Collagenase exposure-induced MMP-1 expression in fibroblast-like synoviocytes**

**Figure S2. Fibroblast-localized MMP-1 expression after bacterial collagenase exposure.** Collagenase exposure for 20mins increases FLS-localized MMP-1 expression (collagenase n=4 gels; vehicle n=2 gels; Wilcoxon test; *p<0.001) quantified by the co-localization of the fibroblast structural protein vimentin (green) and MMP-1 (red). A DAPI label for nuclei is also shown (blue). Images show the maximum projection image of acquired confocal stacks (n=8/gel) throughout the gel. The horizontal lines in the box-and-whisker plot show the first (lower) quartile, median, and third (upper) quartile of the data; whiskers are the minimum and maximum.
